# AMPK Enhances Transcription of Selected Nrf2 Target Genes via Negative Regulation of Bach1

**DOI:** 10.3389/fcell.2020.00628

**Published:** 2020-07-14

**Authors:** Katrin Fischhuber, Manuel Matzinger, Elke H. Heiss

**Affiliations:** Department of Pharmacognosy, University of Vienna, Vienna, Austria

**Keywords:** AMPK, Nrf2, Bach1, gene expression, ARE sites

## Abstract

5′-AMP-activated protein kinase (AMPK) and the transcription factor nuclear factor erythroid 2-related factor 2 (Nrf2) are main players in the cellular adaptive response to metabolic and oxidative/xenobiotic stress, respectively. AMPK does not only balance the rate of fuel catabolism versus anabolism but also emerges as regulator of gene expression. We here examined the influence of AMPK on Nrf2-dependent gene transcription and the potential interplay of the two cellular stress hubs. Using gene expression analyses in wt and AMPKα1 −/− or Nrf2 −/− mouse embryonal fibroblasts, we could show that AMPK only affected a portion of the entire of Nrf2-dependent transcriptome upon exposure to the Nrf2 activator sulforaphane (Sfn). Focusing on selected genes with positive regulation by Nrf2 and either positive or no further regulation by AMPK, we revealed that altered Nrf2 levels could not account for the distinct extent of transactivation of certain Nrf2 targets in wt and AMPK −/− cells (assessed by immunoblot). FAIRE-qPCR largely excluded distinct chromatin accessibility of selected Nrf2-responsive antioxidant response elements (ARE) within the regulatory gene regions in wt and AMPK−/− cells. However, expression analyses and ChIP-qPCR showed that in AMPK−/− cells, levels of BTB and CNC homology 1 (Bach1), a competitor of Nrf2 for ARE sites with predominant repressor function, were higher, and Bach1 also bound to a greater relative extent to the examined ARE sites when compared to Nrf2. The negative influence of AMPK on Bach1 was confirmed by pharmacological and genetic approaches and occurred at the level of mRNA synthesis. Overall, the observed AMPK-mediated boost in transactivation of a subset of Nrf2 target genes involves downregulation of Bach1 and subsequent favored binding of activating Nrf2 over repressing Bach1 to the examined ARE sites.

## Introduction

5′-AMP-activated protein kinase (AMPK) is a heterotrimeric serine/threonine kinase and an important control hub in the cellular energy homeostasis. It is composed of a catalytic α as well as regulatory β and γ subunits, with each subunit occurring in two to three possible isoforms, allowing formation of overall 12 different tissue- and partly substrate- specific trimers (Ross et al., 2016). AMPK’s enzymatic activity is elevated in the presence of high AMP/ATP or ADP/ATP ratios, high calcium levels, or limited glucose supply. Activation is achieved allosterically (by binding of AMP to the γ-, or small molecules to the β- subunit) or covalently via phosphorylation at Thr 172 (reviewed in Steinberg and Carling, 2019). Activated AMPK generally increases catabolic and decreases anabolic activities, leading to increased ATP generation and reduced ATP consumption (Ross et al., 2016; Steinberg and Carling, 2019). Activated AMPK has also been linked to regulation of gene expression, either by direct phosphorylation of transcription factors, histones and epigenetic regulators, or indirectly by modulating the supply of metabolites needed for epigenetic and posttranslational tags (e.g., Bungard et al., 2010; Nieminen et al., 2013; Xu et al., 2014; Salminen et al., 2016; Yang et al., 2016; Marin et al., 2017; Saline et al., 2019; Dziewulska et al., 2020). Like this, AMPK activity influences multiple cellular responses including inflammation and protection from oxidative stress (Salminen et al., 2011; Lin et al., 2017).

The transcription factor nuclear factor erythroid 2-related factor 2 (Nrf2) belongs to the family of the Cap ‘n‘collar basic region leucine zippers and directly regulates more than 200 genes. Among those there are genes involved in the antioxidant defense, drug metabolism and disposition, cell proliferation or lipid metabolism (Chorley et al., 2012). Under basal conditions, Nrf2 activity is low due to its interaction with Kelch-like ECH-associated protein 1 (Keap1) or with β-transducin repeat containing protein 1 (β-TrCP), leading to continuous Nrf2 ubiquitylation and proteasomal degradation (Itoh et al., 1999; Rada et al., 2011). Upon diverse stressful insults and exogenous cues, ubiquitination ceases, Nrf2 accumulates and translocates to the nucleus. There, Nrf2 heterodimerizes with small musculoaponeurotic fibrosarcoma (Maf) proteins and binds to regulatory DNA elements (Tonelli et al., 2018). Those contain so-called antioxidant response elements (ARE), also known as EpRE (electrophile response elements) or CsMBE (CNC-sMaf binding elements) with the consensus sequence [A/G]TGA[G/C]TCAGCA (Otsuki and Yamamoto, 2019). Other members of the CNC family of transcriptional regulators, including the mainly repressive BTB and CNC homology 1 (Bach1), can compete with Nrf2 for those sites and accordingly influence target gene expression (Sun et al., 2004; Dhakshinamoorthy et al., 2005; Chang et al., 2017). Moreover, several additional (post-) transcriptional and post-translational control mechanisms allow Nrf2-dependent gene transcription to receive and integrate cues from different signaling pathways (Tonelli et al., 2018).

Notably, several reports also suggested a crosstalk between Nrf2 and AMPK signaling in a direct or indirect manner. In this line, AMPK was shown to increase Nrf2 abundance by direct phosphorylation, by triggering p62-dependent autophagy of the Nrf2 inhibitor Keap1 or impeding GSK3β/ βTrcP-mediated Nrf2 degradation (Joo et al., 2016; Lee et al., 2016; Lv et al., 2017). Thus, positive cooperativity between AMPK and Nrf2 was proposed (Mo et al., 2014; Zimmermann et al., 2015; Ci et al., 2017), mainly explained by an increased Nrf2 level upon AMPK activation and/or confirmed by examination of one or few selected Nrf2 target genes.

In this study we set out to investigate (i) whether the entire Nrf2-dependent transcriptome is evenly susceptible to signals from AMPK and (ii) whether AMPK may affect Nrf2 target gene expression by means other than Nrf2 stabilization.

## Materials and Methods

### Chemicals and Antibodies

DL-Sulforaphane (Sfn) (#S4441), MG132 (#474787), SBI0206965 (#SML1540-5MG), Actinomycin D (#A1410), Hemin (#H9039) were from Sigma-Aldrich (Vienna, Austria) and dissolved in DMSO (to at least 1000X stocks), stored at −80°C and added to the cells at the indicated concentrations (maximal final DMSO concentration of 0.2%). Antibodies against AMPKα (#2532), Keap1 (#7705), Nrf2 (#12721), α/β-Tubulin (#2148), Lamin (#12586) as well as secondary horseradish peroxidase-coupled antibodies against mouse (#7076) and rabbit (#7074) were purchased from Cell Signaling (Frankfurt am Main, Germany) and used at 1:1,000 dilution for Western Blotting and 1:100 for immunoprecipitation. Anti-Bach1 (#sc-271211) and anti-GFP antibody (#sc-9996) came from Santa Cruz (Heidelberg, Germany), used at 1:300 dilutions for Western Blotting and 1:50 for immunoprecipitation. The α-actin antibody (#69100) was from MP Biologicals (Illkirch, France) and used in a 1:5,000 dilution.

### Cell Culture

Wt and AMPKα1 −/− mouse embryonic fibroblasts (MEFs) (from now on referred to as AMPK−/−) were kindly provided by B. Viollet, and wt and isogenic Nrf2−/− MEFs were kindly provided by T. Kensler. Routinely tested as mycoplasm-free they were cultivated in Dulbecco’s modified essential medium (DMEM; Lonza, Basel, Switzerland), supplemented with 10% (v/v) fetal bovine serum (Gibco^TM^, via Thermo Fisher Scientific, Rockford, IL, United States), 2 mM glutamine (Invitrogen, Invitrogen, Carlsbad, CA, United States), 100 U/mL benzylpenicillin (Invitrogen) and 100 μg/mL streptomycin (Invitrogen) at 37°C and 5% CO_2_. For experiments, cells were seeded in appropriate dishes and grown to 70% confluence before treatment with test compounds as indicated.

### Microarray and Pathway Analysis

Wt and AMPK −/− or Nrf2−/− MEFs were seeded in a density of 0.5 × 10^6^ in 6 cm dishes and treated the next day with DMSO or 5 μM Sfn for 4 h (in three independent biological replicates). In a concentration of 5 μM (although likely supraphysiologically high), the used tool compound Sfn led to reproducible and marked activation of Nrf2- and AMPK signaling in MEF without exerting any sign of cytotoxicity within 24 h. Then RNA was isolated using RNeasy Mini kit (#74104) from Qiagen (Hilden, Germany) according to the manufacturer’s instructions before it was treated with RQ1 DNase (#M6101, Promega, Walldorf, Germany) for removal of DNA contaminants. RNA quality was controlled via common agarose gel electrophoresis and capillary electrophoresis with a Bioanalyzer (Agilent). Microarray analysis was performed with an Affymetrix Clariom^TM^ S Assay for mouse (#902930) from Thermo Fisher Scientific at the Genomics Core Facility of the Medical University Vienna. Microarray data were analyzed using Bioconductor^1^ and R version 3.4.0^2^. Raw data (.cel) files were processed using the Robust Multichip Average (RMA) algorithm to obtain background corrected, quantile normalized and log base 2 transformed data. A non-specific filter was applied prior to hypothesis testing. Probesets with interquartile range (IQR) across the samples on the log base 2 scale of a least 0.5 were included for further analysis (in total 10218 probesets). To identify differentially expressed genes, statistical comparison with Bioconductor- package limma (linear models for microarray data) using an empirical Bayes method for multiple testing correction based on the false discovery rate (FDR) was used to produce adjusted *P*-values. Probesets with an absolute fold change (fc) of at least 2 and a FDR <0.05 between wt and knockout cells were selected as differentially expressed. For subsequent pathway analysis, Advaita Bio’s iPathwayGuide^3^ or DAVID (Database for Annotation, Visualization and Integrated Discovery^4^) tools were used as described in Draghici et al. (2007), Tarca et al. (2009), Donato et al. (2013), Ahsan and Drãghici (2017), and Dennis et al. (2003).

### Real-Time Quantitative Polymerase Chain Reaction (RT-qPCR)

Wt and AMPK −/− or Nrf2−/− were seeded at a density of 0.35–0.5 × 10^6^ in 6 cm dishes and treated the next day with DMSO or 5 μM Sfn for 4 h (in at least three independent biological replicates). RNA was isolated from MEFs either with RNeasy Mini kit (#74104) (RNA used for microarray (see section “Microarray and Pathway Analysis”) and subsequent validation by qPCR) from Qiagen or with peqGOLD Total RNA Kit obtained via VWR (Vienna, Austria) (#12-6834-02; RNA isolations for experiments focusing on the AMPK/Bach1 interplay). RNA integrity was routinely assessed by agarose gel electrophoresis, and quantification was achieved by spectrometric measurements using a NanoDrop 2000 (Thermo Fisher Scientific), also allowing purity check via the A260/A280 ratio (≥2.0). 1 μg of RNA was transcribed into cDNA with High-Capacity cDNA Reverse Transcription Kit (Applied Biosystems^TM^, via Thermo Fisher Scientific), using the included and for the experiment accordingly diluted 10× RT Buffer, 10× RT Random primers, 25× dNTP Mix (100 mM), MultiScribe Reverse Transcriptase (50 U/μl) and RNase inhibitor (N8080119) ordered separately from the same company. The PCR was performed in a 20 μl reaction volume, using the recommended protocol of 25°C for 10 min, 37°C for 120 min, 85°C for 5 min and 4°C for ∞. cDNA was afterward stored at −80°C until further processing. RT-qPCR was performed using Luna^®^ qPCR Universal master mix (#M3003) from New England Biolabs (Frankfurt am Main, Germany). 10× QuantiTect Primer Assays for murine glutamate- cysteine ligase catalytic subunit (*gclc* NM_010295; #QT00130543), NAD(P)H quinone dehydrogenase (*nqo*) 1 (NM_008706; #QT00094367), glutathione *S*-transferase A (*gsta*) 4 (NM_010357; #QT00098987), thioredoxin reductase (*txnrd*; NM_015762; #QT00146272) 1, aldo-keto reductase family 1 member (*akr1*) c14 (NM_134072; #QT00112749), BTB domain and CNC homolog (*bach*) 1 (NM_007520; #QT00105532) and hypoxanthine phosphoribosyltransferase (*hprt*) *1* (NM_013556; #QT00166768) were purchased from Qiagen, used at 1× concentrations and confirmed to work with amplification efficiencies between 96.6 and 102% under our experimental conditions (derived from the respective slope of calibration curves). Custom- synthesized primers for mouse heme oxygenase (*hmox* NM_010442) 1 (fwd: AAGCCGAGAATGCTGAGTTCA, rev: GCCGTGTAGATATGGTACAAGGA; Ying et al., 2019) were ordered from Thermo Fisher Scientific and used at a final concentration of 0.5 μM. Their amplification efficiency was derived from the slope of linear calibration curves plotting Cq against log (template concentration); % amplification efficiency = [10^(–1/*slope*)^] − 1) × 100, determined with only 87.6% and accordingly taken into account in the evaluation of the data (i.e., amounts of target were quantified based on constructed standard curves, divided by the amount of reference and compared to the calibrator sample). *hprt1* was taken as reference gene as it excelled in pilot experiments over other tested reference genes (*actin, 18S, or gapdh*) in the used experimental setups due to its stable expression throughout the employed treatment regimens (as determined by absolute quantification in 10^5^ cells after respective treatment, based on a standard curve established with multiple dilutions of a plasmid carrying the *hprt1* target sequence), comparable expression between all used cell types (wt, AMPK−/− and Nrf2−/− MEF) and its Cq values close to the ones of the investigated target genes. PCR was performed on a Light Cycler^TM^ LC480 using 40 ng cDNA/well, appropriate primers and 1× master mix in a reaction volume of 15 μl in semi-skirted 96-well plates (#72.1979.132) sealed with adhesive foil (#95.1993) from Sarstedt (Nümbrecht, Germany). The cycling protocol contained one denaturation step (10 min at 95°C) and up to 50 amplification cycles (15 s at 95°C, 30 s at 60°C) as well as melting curves between 55 and 95°C. Quality of the amplification was ensured by a single peak in the melting curve, only one amplicon of the desired size on an agarose gel and no amplification in the negative (no template) control. Unless stated otherwise, compiled data were analyzed with the 2^–Δ^ ^Δ^ *^*C*^*_*q*_ method with log transformation, mean centering and autoscoring basically as previously described (Willems et al., 2008).

### Formaldehyde- Assisted Isolation of Regulatory Elements (FAIRE)

To assess free versus histone-bound chromatin we followed the protocol essentially as described in Rodríguez-Gil et al. (2018). Cells were seeded at a density of 5 × 10^6^ in 15 cm dishes, treated the next day with 5 μM Sfn or 0.05% DMSO for 3 h and fixed for 10 min with 0.75% formaldehyde. Formaldehyde was quenched for 5 min with 125 mM glycine, before cells were washed twice with ice- cold PBS and then harvested. Two 15 cm dishes were pooled for each condition to ensure an appropriate amount of chromatin for further workup steps. Cells were lysed with FAIRE lysis buffer (1 × 10^7^ cells/ml; 50 mM HEPES-NaOH [pH 7.5], 140 mM NaCl, 1 mM EDTA [pH 8], 1% Triton X-100; 0.1% Sodium Deoxycholate, 0,1% SDS, 40 μl/ml cOmplete^TM^ (Roche, added right before use) and 2 mM PMSF (added right before use) by carefully pipetting up and down several times and incubating for 10–20 min on ice.

Chromatin was sheared with Covaris S220 sonicator in 130 μl microTUBEs (PN 520045) until a size of 100–300 bp was reached (Duty Factor: 20%, Peak Incident Power: 175, Cycles/Burst: 200, Duration: 60 s, Cycles: 16, Mode: Frequency Sweeping). The sheared chromatin samples were then centrifuged for 15 min at 16,000 *g*, 4°C, to remove cellular debris. Supernatants containing the chromatin, were snap-frozen with liquid nitrogen as 100 μl aliquots and stored at −80°C if necessary. 50 μl of sheared chromatin were used for determining DNA concentration and chromatin fragment size. 70 μl of elution buffer (1% SDS, 100 mM NaHCO3) and 4.8 μl of 5 M NaCl were added to the samples, before they were digested with 2 μl RNase A (10 mg/ml, Thermo Fisher Scientific) for at least 1 h and 2 μl proteinase K (20 mg/ml, New England Biolabs) for at least 1.5 h (routinely checked on an agarose gel). If the chromatin had the desired size, one of the 100 μl aliquots of sheared chromatin was digested for 1 h with 10 μl RNase A and then with proteinase K for 4 h, before de-crosslinking for 6 h at 65°C and isolating genomic DNA with phenol:chloroform:isoamyl alcohol (25:24:1, Sigma-Aldrich) extraction (= total DNA). Another 100 μl chromatin sample was thawed and treated with RNase A for 1 h before DNA was directly isolated from it. Potential inter-crosslinks were removed with a 4 h incubation at 65°C (= free DNA). The ratio of free vs total DNA at specific ARE-sites was accordingly determined with qPCR analysis. Actin primers were used as positive control (pos = open chromatin, used for normalization), and a sequence from the heterochromatin within chromosome 9 was used as negative control (neg = closed chromatin).

Primers for ARE containing sites within the *hmox-1, nqo1* and *gsta4* promotor and controls (*actin, chromosome 9*) were designed with genomic sequences derived from NCBI (assembly: GRCm38.p6) and Primer3web version 4.1.0^5^. Oligos were custom-synthesized and purchased from Thermo Fisher Scientific: hmox-1-1 (fwd: GTGACCCGCGTACTTA AAGG [NC_000074.6, 75093578- 75093597]; rev: CACTCAC TGGTTGTATGCGG [NC_000074.6, 75093648- 75093667]; amplicon length: 90 bp), hmox-1-2 (fwd: GGGACA AAAGGCACAAAGAGC [NC_000074.6, 75089564- 75089584]; rev: GGAAATCACAACTCAGCATTCC [NC_000074.6, 75089647- 75089668]; amplicon length: 105 bp), hmox-1-3 (fwd: GCTGTGCCTTTTCTGCTGAG [NC_000074.6, 75083935- 75083954]; rev: AGGGTTCAGTCTGGAGCAAC [NC_000074.6, 75084023- 75084042]; amplicon length: 108 bp), gsta4 (fwd: CCAGCACAGGAATCGGAGTC [NC_000075.6, 78191806- 78191825]; rev: CCGGGGAGAAGAACAGGTTT [NC_000075.6; 78191885- 78191904]; amplicon length: 99 bp), nqo1 (fwd: AGTCACAGTGAGTCGGCAAA [NC_000074.6; 107403630- 107403649]; rev: GTGGGAAGTCACCTTTGCAC [NC_000074.6; 107403569- 107403588], amplicon length: 81 bp), actin (positive control) (fwd: TGGGGTTTTCTTGGGGATCG [NC_000071.6, 142905511- 142905530]; rev: CCTTCTGAC CCATTCCCACC [NC_000071.6, 142905449- 142905468]; amplicon length: 90 bp), chromosome 9 (negative control; heterochromatin) (fwd: CTCCCACCACACATGTCTCC [NC_ 000075.6, 77687517- 77687536]; rev: CTCAGTCGCATCC ACACTCT [NC_000075.6, 77687587- 77687606]; amplicon length: 105 bp). The *R*^2^ of each primer pair was tested and was between 0.9856 and 1.

### Chromatin Immunoprecipitation (ChIP)

Cells were grown in a density of 10–15 × 10^6^ cells/15 cm dish and treated with 5 μM Sfn or 0.05% DMSO for 3 h. Crosslinking (10 min with 1% formaldehyde) and cell lysis were mainly performed according to the ChIP-IT Express kit (#102026) from Active Motive (Carlsbad, CA, United States), except for using 15 ml instead of 20 ml of the fixation solution and no PBS- washing step between fixation and Glycine-stop solution as well as centrifugation for 4 min at 209 *g*. Isolated nuclei were resuspended in ChIP buffer from the ChIP-IT High Sensitivity^®^ kit (#53040) from Active Motif and incubated on ice for 10 min before shearing the chromatin with Covaris sonicator S220 until a desired size of 200–1,000 bp average was reached (Duty Factor: 10%, Peak Incident Power: 140, Cycles/Burst: 200, Duration: 55 s, Mode: Frequency Sweeping). Chromatin fragments were evaluated for their size and concentrations on an agarose gel. For the immunoprecipitations, 20–30 μg of chromatin were used. IPs were performed according to the ChIP-IT High Sensitivity^®^ kit. Antibodies against RNA Pol II (positive control) and IgG (negative control) were derived from ChIP-IT High Sensitivity^®^ kit, whereas antibodies against Nrf2 and Bach1 were the same ones as used for Western Blot. Functionality of the antibody for IP was ensured by immunoblot analysis after a de-crosslinking step. RNA Pol II antibody was pre-incubated with a bridging antibody (included in ChIP-IT High Sensitivity^®^ Kit) for 1 h, before being applied to the IPs. For qPCR, the same primers (i.e., *hmox-1* (hmox1-1; hmox1-2; hmox1-3), *nqo1* and *gsta4*) were used as for FAIRE. Positive (#71015) and negative control primers (#71012) were purchased from Active Motive and routinely used to evaluate immunoprecipitates obtained with IgG and α-RNA-Pol II, additionally ensuring reliability of the experiment. Combined qPCR data were evaluated according to the fold enrichment method normalizing the values from the immunoprecipitated samples to that from the input DNA (→ % of input) and relating target specific values to IgG values (→ fold enrichment by target specific antibodies over IgG).

### Protein Extraction, SDS Polyacrylamide Gel Electrophoresis and Immunoblot Analysis

Whole cell lysates were obtained as previously described (Heiss et al., 2013). For analysis of nuclear versus cytosolic proteins and the necessary fractionation, cells were first washed with cold PBS and then exposed to buffer 1 [10 mM HEPES pH 7.5, 0.2 mM EDTA, 10 mM KCl, 1% NP40 (IGEPAL^®^), 1 mM DTT, 0.5 mM PMSF, cOmplete^TM^ (Roche)]. Cells were scraped off and transferred into a microtube and incubated for 15 min on ice, with vigorous vortexing every 2–3 min, and centrifuged for 5 min at 11,000 *g*. The supernatant contained the cytosolic fraction. Pellets were washed once with buffer 1 and were then resuspended in buffer 2 [20 mM HEPES pH 7.5, 1.1 mM EDTA, 420 mM NaCl, 1 mM DTT, PMSF and Complete (Roche)], incubated on ice for 15 min with vigorous vortexing every 2–3 min, followed by centrifugation for 5 min at 11,000 *g*. The supernatant contained nuclear proteins. Successful separation of cytosolic and nuclear fractions was routinely validated by immunoblotting of α/β-Tubulin (cytosolic marker) and Lamin (nuclear marker), respectively.

### Immunoprecipitation of Transfected GFP-Nrf2

Wt and AMPK −/− MEF cells were seeded at a density of 0.9 × 10^6^ in 10 cm dishes and transfected with an expression plasmid for GFP-Nrf2 (Addgene #21549), using Lipofectamine LTX and PLUS Reagent (LifeTech) according to the manufacturer’s instructions. After 24 h, corresponding cells were reseeded into 6 cm dishes, treated with MG132 (20 μM) for Nrf2 stabilization and additionally exposed to DMSO or Sfn (5 μM) as indicated. After protein extraction with RIPA buffer (10 mM Tris/Cl pH 7.5, 150 mM NaCl, 0.5 mM EDTA, 0.1% SDS, 1% Triton X-100, 1% Deoxycholate supplemented with: Roche cOmplete^TM^ Mini Protease Inhibitor Cocktail, 1 mM PMSF, 1 mM NaF, 1 mM Na_3_VO_4_, 5 mM sodium butyrate, 2.5 mM MgCl_2_ and 90 μ DNase I prior to use) and sonication, equal total protein amounts (range of 400 μg) were incubated with 25 μL GFP-Trap^®^ magnetic agarose beads (MA GFP-Trap^®^ from Chromotek, Planegg, Germany) and worked up by washing using RIPA buffer and finally by washing with a detergent free buffer (10 mM Tris/Cl pH 7.5; 50 mM NaCl). For elution, beads were incubated in 1× SDS buffer at 95°C for 5 min, and the eluate was used for SDS-PAGE and immunoblot analysis for GFP and Keap1.

### Knockdown of AMPKα1

Wt MEFs were seeded into 12-well plates at a density of 0.75 × 10^5^ cells/well and after 18 h transfected with three different siRNA sequences targeting AMPKα1 (40 pmol each, #LQ-041035-00-0005, from Dharmacon^TM^ via THP Medical Products, Vienna, Austria) or scrambled control siRNA (from Invitrogen) using Lipofectamine RNAimax (Invitrogen) according to the manufacturers’ instructions. After 48 h, cells were lysed and immunoblotted for AMPK, Bach1 and actin.

### Statistical Analysis

Unless stated otherwise, at least three independent biological replicates (i.e., independent cell batches/passages in independent experiments/stimulations) were performed for all experiments. The bar graphs depict mean + SD (standard deviation) or 95% CI (confidence intervall). Groups were compared via Student’s *t*-test or ANOVA using GraphPad Prism 6 software as indicated in the figure legends. Differences considered as significant (*P* < 0.05) are labeled with asterisk (^∗^) within graphs.

## Results

### AMPK Only Affects the Expression of a Subset of Nrf2 Target Genes

**FIGURE 1:**
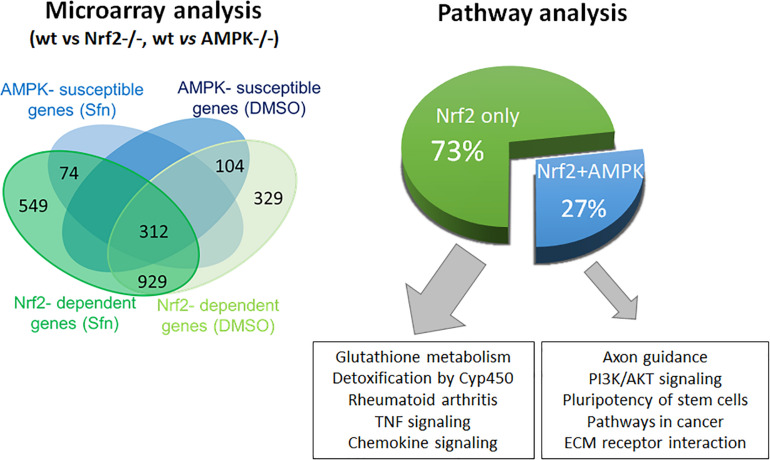
AMPK influences expression of only a subset of the Nrf2-controlled genes. Microarray data from wt and Nrf2 −/− or AMPK −/− were evaluated with regard to statistically significant different (5% FDR, fc > 2) expression of Nrf2-regulated genes between wt and AMPK−/− MEFs and categorized by subsequent pathway analyses.

**FIGURE 2:**
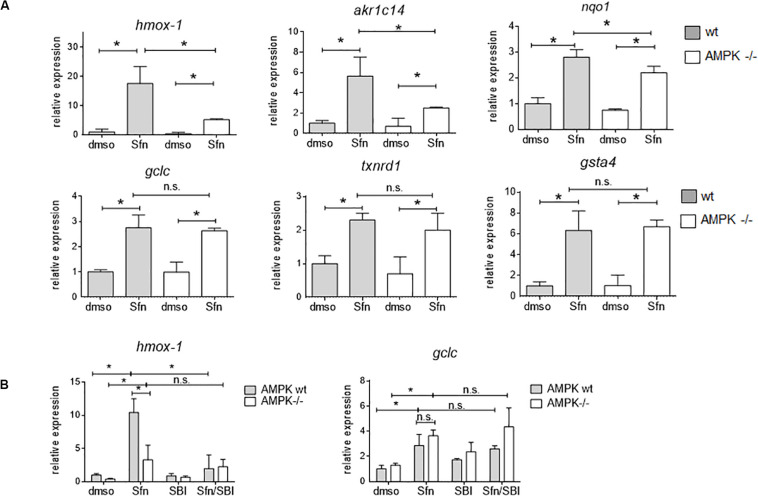
AMPK-mediated boosted transcription of selected Nrf2 target genes is confirmed by qPCR analysis. **(A)** Wt and AMPK −/− MEFs were treated with DMSO (0.1%) or sulforaphane (Sfn, 5 μM) for 4 h before RNA was isolated, reversely transcribed and subjected to qPCR analysis for *hmox1, akr1c14, nqo1, gclc, txnrd1* and *gsta4* as indicated (*hprt1* as reference gene). **(B)** Experimental set up as in panel **(A)**, except that cells were (co)-treated with the AMPK inhibitor SBI0206965 (SBI, 30 μM) as indicated. **(A,B)** Bar graphs present the mean + 95% CI (*n* = 3–4; *P ≤ 0.05, two-way ANOVA, Tuckey post-hoc test; ns: not significant).

In order to assess the influence of AMPK on Nrf2-dependent target genes we performed microarray analyses. We treated wt, Nrf2 −/− and AMPK−/− MEFs with sulforaphane (Sfn), a natural compound well known for activation of Nrf2 and also AMPK signaling (Magesh et al., 2012; Choi et al., 2014). After 4 h, RNA was extracted, transcribed to cDNA, probed on a murine whole genome Affymetrix Chip and statistically evaluated for different expression between wt and knockout cells (see Supplementary Table 1). Transcripts that showed significantly different expression between wt and Nrf2−/− cells (FDR < 0.05; >2-fold differences in expression) were considered Nrf2-dependent. Those were then tested for different expression between wt and AMPK −/− cells. From 1,807 (directly or indirectly) Nrf2-regulated genes (929 genes differently regulated in DMSO- and Sfn-treated cells, 329 genes differently regulated only in DMSO- treated setting and 549 genes differently regulated only in Sfn-treated setting), only 490 genes (27%) appeared to be susceptible to the action/presence of AMPK (Figure 1 and Supplementary Table 2). Pathway analysis using Advaita or DAVID tools consistently showed a trend that genes belonging to the category of glutathione metabolism, xenobiotic detoxification or inflammation were not susceptible to AMPK presence. Nrf2-dependent genes with impact on PI3K/Akt signaling, on pathways within cancer or pluripotent stem cell signaling or ECM receptor interaction were responsive to AMPK signaling (Supplementary Figure 1). Notably, Nrf2 could hereby either positively (e.g., *hmox-1, gclc, gsta4, akr1c14*) or negatively (e.g., *dusp4, sulf1*) influence expression of respective genes and in turn was either supported (e.g., *hmox-1*) or counteracted (e.g., *sulf1*) by AMPK. For confirmation of microarray data by qPCR analyses and further in-depth examination throughout this work we selected genes that were directly regulated by Nrf2 binding in their regulatory regions [according to published ChIP data (Malhotra et al., 2010; Chorley et al., 2012; Namani et al., 2019), primarily or only inducible by Nrf2 in response to Sfn (no change in Nrf2 −/− cells) and either boosted or unaffected by the presence of AMPK. QPCR analyses could corroborate *hmox1*, *nqo-1*, and *akr1c14* as AMPK-sensitive genes, as their induction upon Nrf2 activation by Sfn was significantly reduced in AMPK −/− cells. The AMPK-responsiveness of *hmox-1* and *nqo1* is in line with previous reports (Mo et al., 2014; Zimmermann et al., 2015). *Gsta4, gclc*, and *txnrd1* showed a comparable extent of transactivation in wt and AMPK−/− cells (Figure 2A). The degree of AMPK-(in)dependence was further confirmed by employment of the AMPK inhibitor SBI0206965 (SBI) (Dite et al., 2018), as exemplified by impaired induction of *hmox1* but not that of *gclc* upon SBI treatment (Figure 2B). Nrf2-dependent induction of all investigated genes was double-checked by qPCR analysis in Sfn-treated wt and Nrf2−/− MEFs (Supplementary Figure 2; Matzinger et al., 2020). Overall, AMPK seems to regulate expression of only a subset of the Nrf2-dependent transcriptome.

### Nuclear Nrf2 or Keap1 Levels Are Not Different Between Wt and AMPK −/− Cells

**FIGURE 3:**
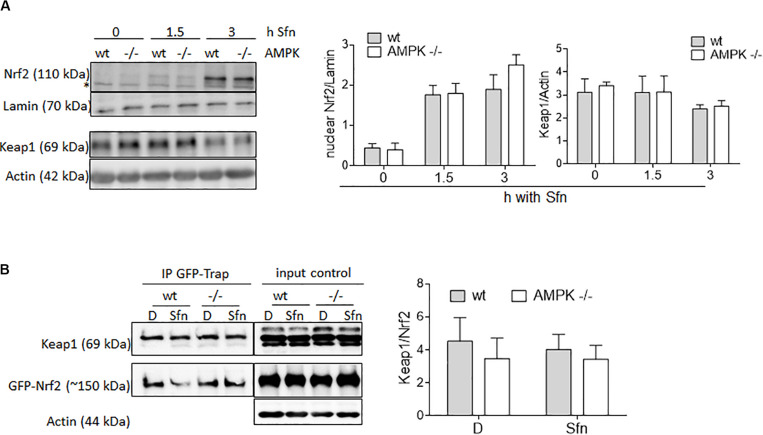
Wt and AMPK−/− MEFs do not differ in abundance of nuclear Nrf2, Keap1 levels, or Nrf2/Keap1 interaction. **(A)** Wt and AMPK −/− MEFs were treated with DMSO (0.1%) or Sfn (5 μM) for the indicated periods of time prior to fractionation of cytosolic and nuclear proteins. Both fractions were subjected to immunoblot analyses for Nrf2 or Lamin and Keap1 or Actin, respectively. Representative blot pictures as well as compiled densitometric data of three independent experiments are depicted (*n* = 3, mean + SD). The “*” depicts an unspecific band recognized by the used α-Nrf2 antibody in MEF lysates. **(B)** Wt and AMPK−/− cells were transiently transfected with an expression plasmids GFP-Nrf2 and upon Nrf2 stabilization by MG132 treated with DMSO (D, 0.1%) or Sfn (5 μM) as indicated for 2 h. After cell lysis, GFP-Nrf2 was pulled down by a GFP-trap, loaded on a gel and probed for GFP and Keap1. Prior to pulldown, an aliquot of the lysate was removed to serve as input control. Representative blot pictures as well as compiled densitometric data of three independent experiments are depicted.

AMPK activity has been reported to stabilize Nrf2, mainly due to an impaired GSK3β/βTrCP-triggered degradation or increased p62-mediated autophagy of Keap1 (Lee et al., 2016; Lv et al., 2017). Therefore, we wondered whether different amounts of nuclear Nrf2 could possibly also account for the uneven extent of transactivation of selected Nrf2 target genes between wt and AMPK −/− cells, likely due to context/affinity-dependent distinct ARE occupancy by changing amounts of Nrf2. However, a comparison between nuclear fractions of wt and AMPK −/− MEFs upon Sfn treatment did not reveal any obvious difference in Nrf2 abundance (Figure 3A). Also, Keap1 levels (Figure 3A) as well as the Nrf2/Keap1 interaction (Figure 3B) were unaltered between wt and AMPK −/− cells in our cell system after Sfn treatment. This data set indicates that different levels of nuclear Nrf2 cannot markedly account for the differences in Nrf2 target gene expression seen between Sfn-treated wt and AMPK−/− cells. Moreover, Sfn leads to stabilization of Nrf2, however, irrespective of AMPK.

### Wt and AMPK−/− Cells Do Not Markedly Differ in the Local Chromatin Opening at Selected ARE Sites

**FIGURE 4:**
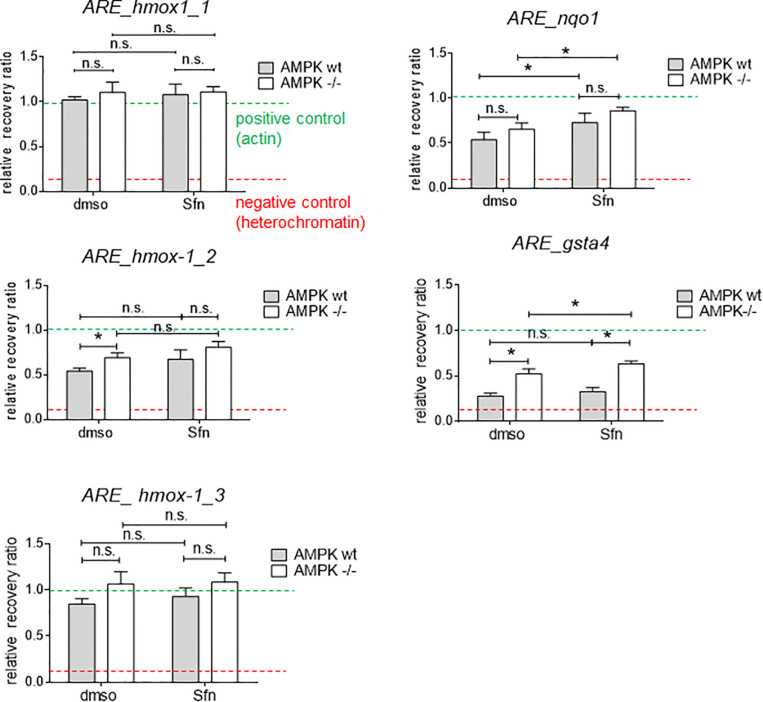
Wt and AMPK −/− cells hardly differ in the chromatin opening at selected ARE sites. Cells were treated with DMSO (0.05%) or Sfn (5 μM) as indicated for 3 h. After crosslinking and chromatin shearing, free and histone-bound DNA were subjected to FAIRE-qPCR analysis for selected sites as described in detail in section “Materials and Methods.” As positive control a regulatory region within actin gene was used (mean chromatin opening of 1.0; green dotted line), and heterochromatin served as negative control for little chromatin accessibility (red dotted line); hmox-1-1,2,3: ARE sites within proximal promoter or within distal enhancer regions E1 and E2; nqo1, gsta4: ARE sites within proximal promoters. Bar graph depicts compiled data of three independent experiments. (mean + 95% CI; *n* = 3; *P ≤ 0.05, two-way ANOVA, Tuckey post-hoc test; ns: not significant).

AMPK is known to influence chromatin accessibility e.g., via modulated histone acetylation (e.g., Gongol et al., 2018). Notably, AMPK −/− cells displayed higher global acetylation of selected lysine residues on histone 3 (K14,18, 27, and 56) (Supplementary Figure 3), possibly causing a dilution effect of Nrf2 by more accessible binding sites in the knockout cells, as previously outlined (Brewster et al., 2014; Rydenfelt et al., 2014). We therefore analyzed local chromatin opening at different ARE sites in regulatory regions of *hmox-1* (sites 1–3 located in promoter and enhancer E1 and E2 regions), *nqo1*, and *gsta4* in wt and AMPK −/− cells (Alam and Cook, 2003; Alam et al., 2004; Figure 4). According to results from FAIRE-qPCR experiments, there were no striking or consistent differences in the opening state of almost all tested regulatory regions between wt and AMPK−/− cells. The tested ARE sites appeared relatively accessible (based on the opening state of heterochromatin and actin as negative and positive controls, respectively) already in the basal (DMSO treated) state and partly showed moderate tendency to increased opening upon Sfn treatment. Only for the tested ARE site in *gsta4*, AMPK −/− cells clearly showed increased accessibility in both the DMSO- and Sfn-treated condition. However, as *gsta4* is an AMPK-unresponsive gene, higher chromatin opening and a potential aggravated competition for limited nuclear Nrf2 could not provide a consistent explanation for the observed reduced transactivation of a subset of Nrf2 target genes in Sfn-treated AMPK−/− cells.

### AMPK −/− Cells Show Reduced Nrf2 Over Bach1 Enrichment at ARE Sites and Elevated Bach1 Levels

**FIGURE 5:**
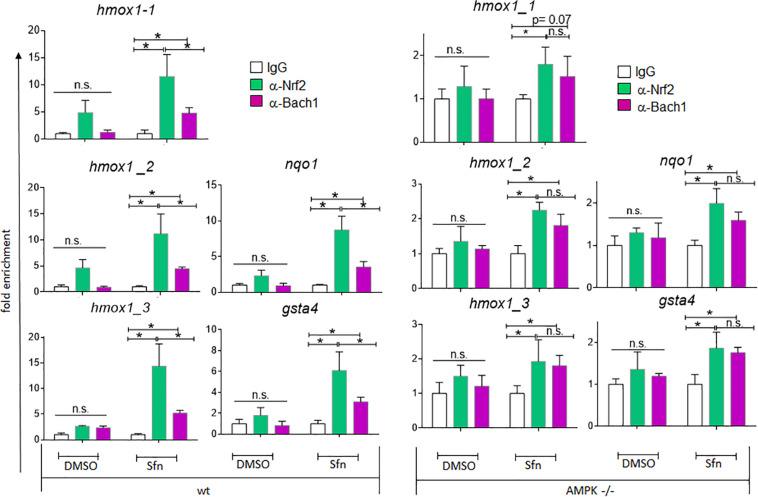
Wt and AMPK−/− cells show distinct relative ratios of Bach1 and Nrf2 at ARE sites. Wt and AMPK−/− cells were treated with Sfn (5 μM) or 0.05% DMSO for 3 h. ChIPs were performed as described in section “Materials and Methods” using antibodies specific for Nrf2 or Bach1 and IgG (negative control), before isolating DNA for qPCR analysis of the indicated ARE-sites within *hmox1*, *nqo1* and *gsta4* genes. The bar graphs depict compiled mean fold enrichment + 95% CI (*n* = 3) of Nrf2 (green) and Bach1 (red) at the respective sites, calculated in reference to IgG (white) immunoprecipitates (*P ≤ 0.05; ANOVA, Tuckey post-hoc test).

**FIGURE 6:**
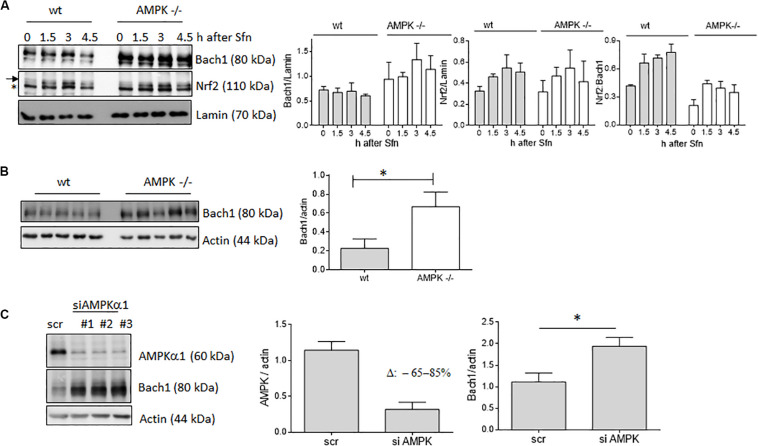
AMPK exerts a negative influence on Bach1 levels. **(A)** Wt and AMPK−/− cells were treated with Sfn (5 μM) for the indicated periods. Then, nuclear fractions were probed for Nrf2, Bach1 and Lamin. Representative blots are depicted together with the densitometric analyses of Nrf2/Lamin, Bach1/Lamin as well as Nrf2/Bach1 of three independent biological replicates. The “*” depicts an unspecific band recognized by the used α-Nrf2 antibody in MEF extracts. **(B)** Lysates of wt and AMPK−/− cells were immunoblotted for Bach1 and actin. The bar graph depicts the densitometric analysis of five independent experiments (mean + SD; Student’s *t*-test, α = 0.05, *P ≤ 0.05). **(C)** Wt cells were transiently transfected with either 40 pmol of scrambled siRNA or siRNA directed against AMPKα1 (three different sequences #1–3). After 48 h lysates were probed for AMPKα, Bach1 and actin. Representative blots and compiled densitometric analyses of three independent experiments (for sequence #1) are depicted (mean + SD; Student’s *t*-test, α = 0.05, *P ≤ 0.05).

**FIGURE 7:**
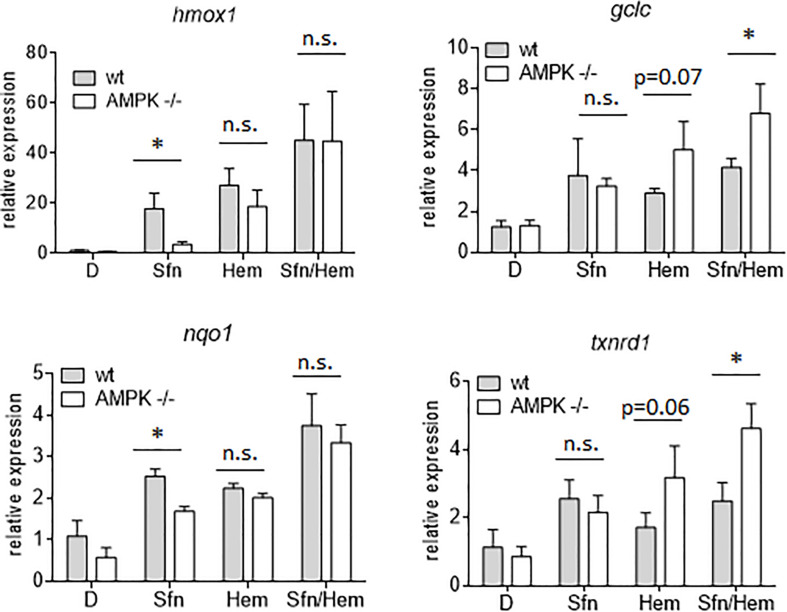
Inhibition of Bach1 by hemin annihilates the distinct Nrf2 target gene expression between wt and AMPK−/− cells. Wt and AMPK −/− MEFs were treated with DMSO (0.1%), Sfn (5 μM) or hemin (Hem, 20 μM) as indicated. After for 4 h RNA was isolated, reversely transcribed and subjected to qPCR analysis for *hmox1*, *gclc, nqo1, txnrd 1* or *hprt* as reference gene (*n* = 3, mean + SD; two-way ANOVA, Tuckey post hoc test; *P ≤ 0.05).

In addition to nuclear Nrf2 abundance and chromatin accessibility, expression of ARE-regulated genes can depend on the competitive binding of activating Nrf2 and repressing Bach1. We therefore performed ChIP-qPCR experiments to examine the relative binding of Nrf2 and Bach1 at selected ARE sites in wt and AMPK−/− cells. Performed in three independent biological replicates they consistently showed that both Nrf2 and Bach1 get enriched at the examined sites upon Sfn treatment. In wt cells, Nrf2 hereby excelled Bach1 enrichment at all investigated ARE sites (evident in ratios of 2.0–2.5 to 1 of mean enriched Nrf2 to Bach1), irrespective of the presumable AMPK susceptibility of the regulated genes. In contrast, in AMPK−/− cells Nrf2 enrichment at the investigated sites could hardly top that of Bach1 (ratio 1.0–1.2 to 1) (Figure 5). Notably, fold enrichment was generally lower for both Nrf2 and Bach1 in AMPK-than in wt cells. This was also true for other tested factors in these two cell types (data not shown), suggesting a cell-inherent feature of AMPK−/− cells, possibly due to universal dilution effects by the overall higher acetylated entire chromatin (see Supplementary Figure 3). The revealed higher Bach1 to Nrf2 ratio at ARE sites in Sfn-treated AMPK−/− cells was also reflected at the level of nuclear abundance of the two competitors (Figure 6A), in line with the overall higher Bach1 expression in AMPK−/− cells (Figure 6B). The negative correlation between AMPK and Bach1 expression consolidated in knockdown and overexpression approaches: Knockdown of AMPKα1 in wt cells via three different specific siRNA sequences (by 65–85%) led to significantly higher Bach1 levels (Figure 6C). In reverse, expression of AMPK (GFP-AMPKα) in AMPK −/− cells resulted in a reproducible reduction of Bach1 protein (Supplementary Figure 4). To see whether the different Bach1 levels could truly mediate the distinct impact of AMPK on selected Nrf2 target genes, we made use of hemin, a known inhibitor of Bach1 (Zenke-Kawasaki et al., 2007). Treatment with hemin or cotreatment with Sfn/hemin enhanced *hmox1, txnrd1, nqo1* and *gclc* expression. Notably, *hmox1* and *nqo1* induction was hereby comparable between wt and AMPK−/− cells (Figure 7). In contrast, *gclc* and *txnrd1* induction was reproducibly higher in AMPK −/− than wt cells (to a significant extent in the hemin/Sfn condition), opposing the picture seen after exposure to the Nrf2 activator Sfn alone (with comparable induction of *gclc* and *txnrd1*, but lower *hmox1* and *nqo1* induction in −/− than in wt cells). Overall, AMPK deficiency is correlated with elevated Bach1 levels and a generally higher ratio of ARE-bound Bach1 to Nrf2. Apparently, this relative increase in bound Bach1 results in reduced transactivation of certain ARE sites, conferring AMPK-responsiveness to the respectively controlled genes.

### AMPK Negatively Regulates *Bach1* Transcription

**FIGURE 8:**
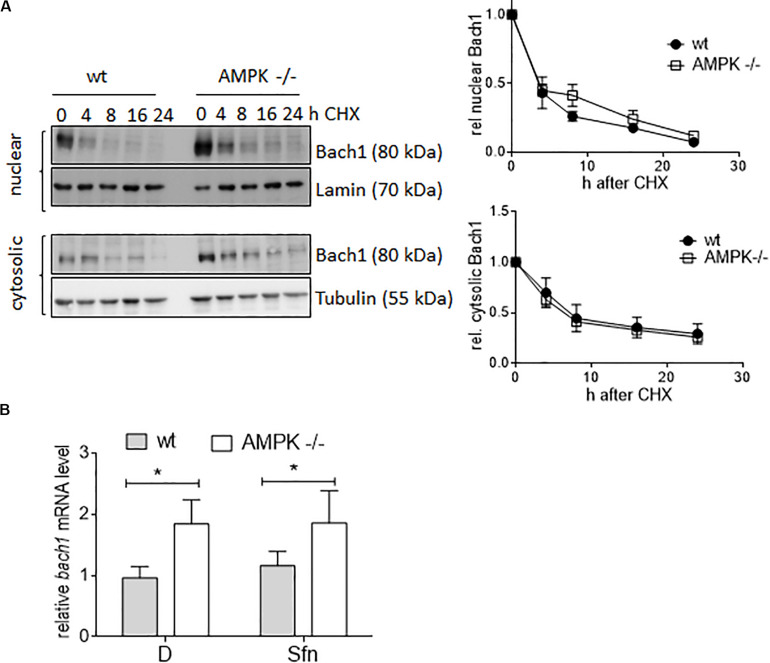
AMPK regulates Bach1 at the transcriptional level. **(A)** Wt and AMPK−/− cells were treated with 10 μM cycloheximide for the indicated periods of time. Nuclear and cytosolic fractions were then probed for Bach1 and actin. Representative blots of three performed experiments are depicted together with compiled densitometric data showing the decay of Bach1 over time. **(B)** Wt and AMPK−/− cells were treated with DMSO (D, 0.1%) or Sfn (5 μM) for 4 h before *bach1* mRNA levels were determined by qPCR (*hprt1* as reference gene) (*n* = 3, mean + SD; two-way ANOVA, Tuckey post hoc test; *P ≤ 0.05).

Next, we aimed at providing first molecular details underlying the uncovered negative correlation between AMPK and Bach1. Monitoring the decay of cytosolic or nuclear Bach1 upon treatment with cycloheximide showed that Bach1 displayed comparable half-life in wt and AMPK −/− cells (Figure 8A). In contrast, both DMSO and Sfn-treated AMPK −/− cells clearly showed elevated *bach1* mRNA expression compared to wt cells, and Sfn did not markedly affect *bach1* mRNA within 4 h (Figure 8B). In addition, the AMPK inhibitor SBI0206965 significantly led to increased mRNA *bach1* expression level over time in wt cells, an effect that was markedly blunted in AMPK −/− cells (Supplementary Figure 5A). Half-life of *bach1* mRNA was not markedly different between wt and AMPK−/− cells (Supplementary Figure 5B). Thus, AMPK leads to reduced Bach1 abundance likely due to diminished mRNA synthesis.

## Discussion

**FIGURE 9:**
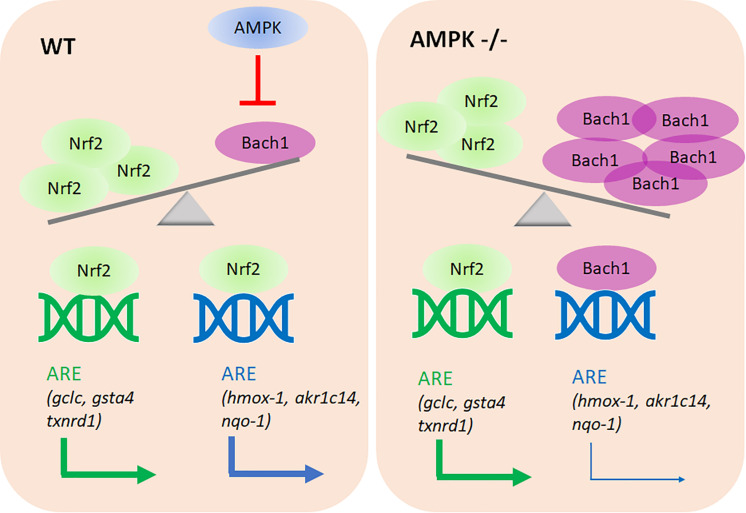
Proposed model of the AMPK/Bach1-mediated selective modulation of ARE/Nrf2-dependent gene expression. AMPK negatively regulates Bach1 expression (at the transcriptional level), leading to de-repression of Nrf2-dependent ARE sites in the regulatory regions of a distinct subset of target genes.

The main novel findings of this study are: (i) AMPK controls only a subset within the Nrf2-dependent transcriptome. (ii) Altered Nrf2 levels or altered accessibility of regulatory ARE sites do not account for the observed differences in target gene transcription between the used wt and AMPK −/− cells. (iii) Rather, AMPK presence/activity ensures reduced Bach1 abundance with preferential Nrf2 over Bach1 binding to regulatory ARE sites, and finally stronger transactivation of selected target genes. (iv) AMPK negatively controls *bach1* mRNA expression (see Figure 9).

So far AMPK activity had mainly been reported to enhance expression of selected Nrf2 target genes, both in a physiological and pathophysiological setting (e.g., Mo et al., 2014; Zimmermann et al., 2015; Finley, 2018). Taking an unbiased look on AMPK’s influence on the entire Nrf2-dependent transcriptome via microarray has now revealed that AMPK can either support, oppose or not affect the activating or repressing effect of Nrf2 on gene expression under basal (DMSO) and activated (Sfn-treated) conditions. Thus, the picture of the interplay between AMPK and Nrf2 signaling seems to be far more complex than deducible from previous studies. AMPK infers a dichotomy onto transactivation of Nrf2-regulated genes: for some genes, activation of Nrf2 already suffices to modulate their expression to a given extent, while others are controlled by Nrf2, however, still susceptible to the cellular energy status and AMPK signaling.

For most selected AMPK-responsive and AMPK-unaffected genes, FAIRE analyses indicated comparable chromatin opening of the ARE sites in their regulatory regions in wt and AMPK−/− cells, despite the known impact of AMPK activity for histone acetylation (Gongol et al., 2018) and an increased global histone acetylation in the used AMPK −/− cells compared to wt cells. Notably, examined ARE sites appeared to be within relatively loosely packed chromatin throughout all tested conditions. This is in line with a previous report on *hmox1* ARE sites (Alam et al., 2004), referring to constant hyperacetylation of associated histones. Persistent access to regulatory regions of Nrf2-responsive genes may enable the cell to promptly respond to stressors with transcription of detoxification genes without the need for extensive chromatin rearrangement. Transcription of those genes could be simply switched on and off by recruiting corresponding activators or repressors.

A striking difference between corresponding ARE sites in wt and AMPK−/− cells arose in their relative occupancy with activating Nrf2 or repressing Bach1 in response to Sfn, with an overall higher Nrf2 to Bach1 ratio in wt than in −/− cells. The underlying ChIP-qPCR experiments may call for some caution, as they directly compare enrichment efficiencies obtained with two different antibodies leaving the risk of distinct performance in the immunoprecipitation step, which, however, is likely to affect wt and AMPK−/− cells to the same extent. Levels of nuclear Nrf2 upon activation (here by Sfn) were not different in wt and AMPK−/− cells, which is consistent with our previous observations (Zimmermann et al., 2015; Matzinger et al., 2020), and could therefore not directly account for the varying Nrf2 to Bach1 ratio. In contrast, *bach1* mRNA and Bach1 protein levels showed a clear inverse correlation with AMPK presence in DMSO and Sfn-treated cells. Notably in this context, previous studies already indicated that activation of regulatory ARE sites can preferentially occur either by Nrf2 activation or Bach1 de-repression (Reichard et al., 2007; Miyazaki et al., 2010; Emter and Natsch, 2015). It therefore may be speculated that AMPK-responsive genes have ARE sites with strong dependence on de-repression of Bach1. In line with this theory, transcription of *hmox1* and *nqo1*, reported to be strongly repressed by Bach1 (Dhakshinamoorthy et al., 2005; Ishikawa et al., 2005; Reichard et al., 2007, 2008), showed susceptibility to AMPK when induced by Sfn, and presence of the Bach1 inhibitor hemin allowed similar induction of *hmox1* and *nqo1* between wt and AMPK−/− cells. *Gclc* and *txnrd1* induction, predominantly controlled by activated Nrf2 rather than Bach1 (Reichard et al., 2007), were similar in wt and AMPK−/− cells upon Sfn exposure. This data needs to be complemented by closer examination of additional genes for further unequivocal corroboration of the hypothesis. What also remains to be resolved in sufficient detail is why only some ARE sites are finally more responsive to Nrf2 activation than Bach1 repression despite a comparable ratio of bound Nrf2:Bach1 throughout all tested ARE sites. Very likely, the number and exact sequence of ARE sites in the regulatory regions of a gene play a role (Reichard et al., 2007; Raghunath et al., 2018; Otsuki and Yamamoto, 2019). Moreover, we recently have uncovered AMPK-dependent phosphorylation of Nrf2 at three serine residues in living cells (Matzinger et al., 2020). Interestingly, this post-translational modification conferred Nrf2 with a boosted transactivation potential for almost the same target genes that are also enhanced in wt versus AMPK−/− cells. Only *nqo1* showed a conflicting result between these studies, possibly indicating that this gene is a borderline case for regulation by the AMPK/Bach1 axis (supported by the rather moderate difference in induction between wt and AMPK−/− cells seen in Figure 2). Nonetheless, it remains plausible that Nrf2 phosphorylation is necessary for ARE binding by Nrf2 and overriding Bach1 repression, most likely in a context-dependent manner. This issue deserves further investigation in the future, also with a special eye on the dynamics of Nrf2 and Bach1’s ARE binding, their mutual regulation, competitive binding to coactivators or co-repressors, and functional and genetic classification of the regulated genes (Dhakshinamoorthy et al., 2005; Jyrkkänen et al., 2011; Lignitto et al., 2019). As AMPK influences *bach1* mRNA expression the question for the involved transcription factor(s) and regulators is prompted. One promising candidate deserving further examination is SP1 as it positively affects *bach1* transcription (Sun et al., 2001), is susceptible to negative regulation by AMPK as shown by others (Hann et al., 2013; Zhao et al., 2016) and in our pilot experiments (data not shown).

Although revealing several novel and intriguing aspects of the crosstalk between AMPK, Bach1 and Nrf2, the presented data still require additional proof for general applicability as they focused on the detailed investigation of only few selected genes with positive regulation by Nrf2 and AMPK, the use of murine embryonal fibroblasts with α1 knockout and only one Nrf2 activator. Future studies are therefore warranted, making use of other cell or model systems, employing holistic ChIP-Seq and RNA-Seq approaches, and elaborating on more extensive pathway analyses upon treatment with chemically/pharmacologically different Nrf2/AMPK activators, optimally also with a higher number of biological replicates. Nonetheless, the discovered link between AMPK and Bach1 as well as the resulting selective influence on Nrf2 target gene expression are compelling and touch existing data. Likewise, Bach1 also contributed to the expression of only selected Nrf2 target genes in endothelial cells under hypoxic conditions (Chapple et al., 2016) which, in turn, are known to influence AMPK activity (e.g., Mungai et al., 2011; Sato et al., 2017; Xue et al., 2017). Moreover, Bach1 levels are elevated during aging (Davies and Forman, 2019), in metastatic lung tumors (Lignitto et al., 2019; Wiel et al., 2019) or triple negative breast tumors with concomitant mitochondrial dysfunction (Lee et al., 2019), all events also partly connected with AMPK- (Yan et al., 2015; Weir et al., 2017; Herzig and Shaw, 2018) and/or Nrf2 activity (Dinkova-Kostova and Abramov, 2015; Zhang et al., 2015; Rojo de la Vega et al., 2018; Silva-Palacios et al., 2018). Thus, these exemplary issues strongly advocate for a closer look into the interplay between the cellular sensors and executors of the oxidative/xenobiotic and metabolic stress response, which likely will uncover additional layers of the finetuned regulation of the cellular stress resilience.

## Data Availability Statement

All datasets generated for this study are included in the article/Supplementary Material.

## Author Contributions

KF and EH designed the experiments. KF, MM, and EH performed the experiments and analyzed the data. EH conceived and supervised the study as well as secured funding. KF and EH drafted the manuscript. All authors read, revised, and agreed to the manuscript.

## Conflict of Interest

The authors declare that the research was conducted in the absence of any commercial or financial relationships that could be construed as a potential conflict of interest.

## Footnotes

1 www.bioconductor.org

2 www.r-project.org

3 https://www.advaitabio.com/ipathwayguide

4 https://david.ncifcrf.gov/

5 http://primer3.ut.ee/
