# Supplementary material for: AMPK Enhances Transcription of Selected Nrf2 Target Genes via Negative Regulation of Bach1
Source: Front Cell Dev Biol. 2020 Jul 14;8:628. doi: 10.3389/fcell.2020.00628 (PMC7372114; doi:10.3389/fcell.2020.00628)
Supplement: Supplementary file 1 [file Image_1.pdf]

Supplemental Material

AMPK enhances transcription of selected Nrf2 target genes via negative regulation of Bach1

Katrin Fischhuber[1], Manuel Matzinger[1,+] and Elke H Heiss[1]*

[1]Department of Pharmacognosy, University of Vienna, Vienna, Austria

Supplemental Table 1: Gene expression data from wt and Nrf2-/ or AMPK -/- MEFs after treatment (4h) with DMSO or Sfn (5 µM) as obtained from an Affymetrix Clariom™ S Assay and subsequent statistical analysis

Supplemental Table 2: List of Nrf2-regulated genes and Nrf2-and AMPK-regulated genes in DMSO- and Sfn-treated MEF

Supplemental Materials and Methods

Expression of AMPK in AMPKα1-/- MEFs

AMPK-/-MEFs were seeded into 12-well plates and transfected with an expression plasmid for eGFP (Clontech) or GFP-AMPKα1 (pEGFP-C1-PRKAA1 #30305 from Addgene) using Lipofectamine LTX and Plus reagent according to the manufacturers' instructions. After 42 hours, cells were lysed and probed for Bach1, GFP or actin.

Supplemental Figures

(A)

**DAVID**	*p*-value		**Advaita**	*p*-value	
**glutathione metabolism**	1.7 e-4		**axon guidance**	4.4 e-5	8.7 e-5
**focal adhesion**	6.5 e-4		**focal adhesion**	2.1 e-4	0.07
**PI3K-Akt signaling pathway**	3.4 e-4	5.9 e-1	**chemokine signaling pathway**	2.2 e-4	
basal cell carcinoma	4.4 e-4	4.3 e-1	**glutathione metabolism**	6.8 e-4	
**ECM receptor interaction**	5.0 e-4	5.0 e-1	**rheumatoid arthritis**	0.001	
**pathways in cancer**	5.8 e-4	5.1 e-1	**malaria**	0.002	0.06
**microRNAs in cancer**	5.9 e-4		central carbon metabolism in cancer	0.002	
**metabolism of xenobiotics by Cyp450**	6.1 e-3		**PI3K-Akt signaling pathway**	0.003	0.07
**axon guidance**	8.0 e-4	5.0 e-1	**microRNAs in cancer**	0.004	
**glucosaminoglycan biosynthesis**	8.5 e-3		**pathways in cancer**	0.004	0.01
**signaling pathways regulating pluripotency**	1.3 e-2	4.5 e-1	toxoplasmosis	0.005	
drug metabolism by Cyp450	1.8 e-2		**proteoglycans in cancer**	0.007	
**malaria**	1.8 e-2		**glycosaminoglycan biosynthesis**	0.007	
**proteoglycans in cancer**	1.9 e-2		platinum drug resistance	0.010	
**chemokine signaling pathways**	2.0 e-2		**signaling pathways regulating pluripotency**	0.011	0.02
**TNF signaling pathway**	3.9 e-2		fatty acid elongation	0.012	
**rheumatoid arthritis**	4.7 e-2		panthotenate and CoA biosynthesis	0.014	
Rap1 signaling pathway	5.1 e-2	6.1 e-1	Hif signaling pathway	0.019	
melanogenesis	5.3 e-2		Raf signaling pathway	0.019	
chemical carcinogenesis	6.3 e-2		prion signaling	0.020	
cAMP signaling	7.3 e-2	7.2 e-1	**TNF signaling pathway**	0.024	
calcium signaling	7.7 e-2		**metabolism of xenobiotics by Cyp 450**	0.026	
renin angiotensin system	8.0 e-2		**ECM receptor interaction**	0.027	0.05
bladder cancer	8.0 e-2		synthesis of unsaturated fatty acids	0.027	
Hippo signaling pathway	9.0 e-2		tight junctions	0.027	

(B)

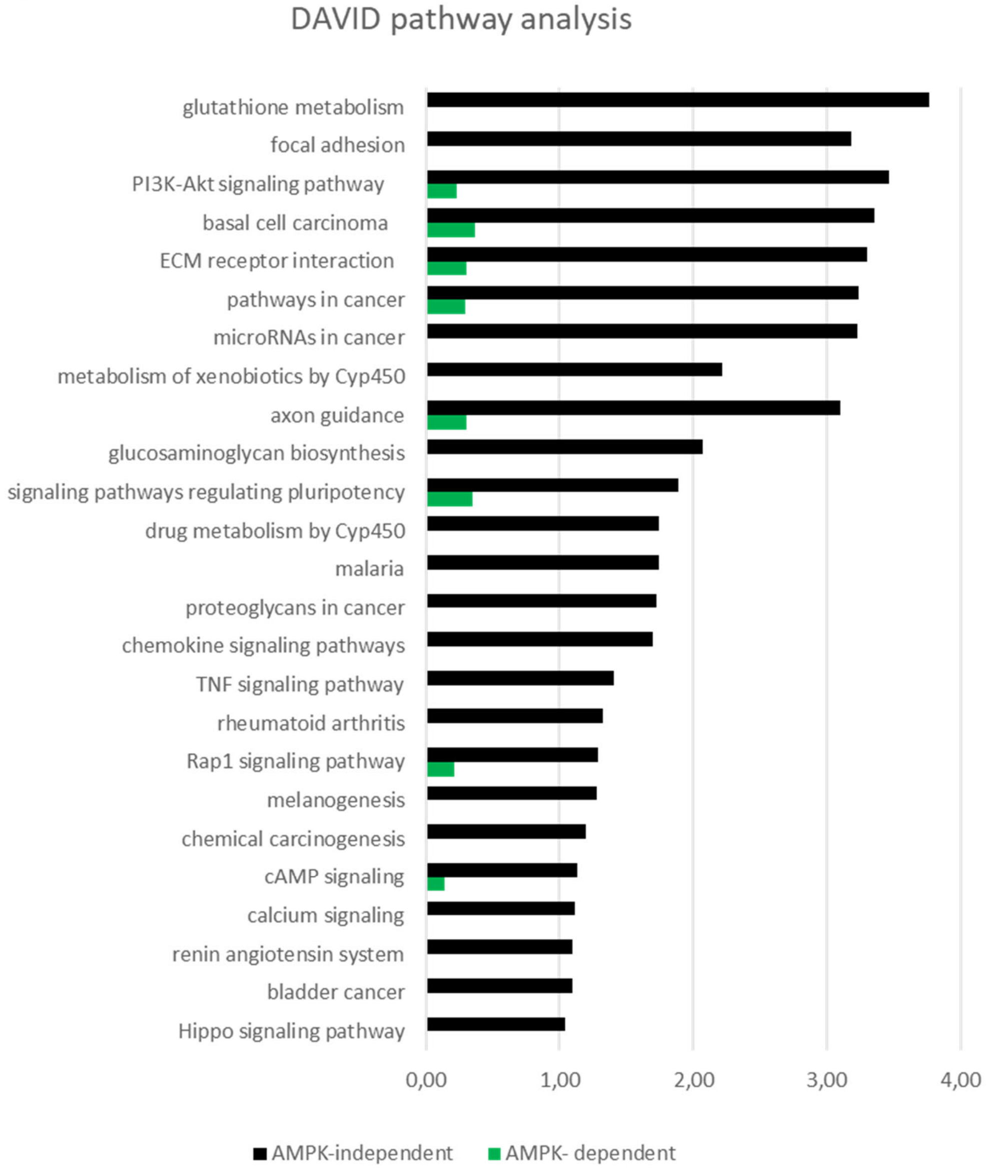
DAVID pathway analysis
glutathione metabolism
focal adhesion
PI3K-Akt signaling pathway
basal cell carcinoma
ECM receptor interaction
pathways in cancer
microRNAs in cancer
metabolism of xenobiotics by Cyp450
axon guidance
glucosaminoglycan biosynthesis
signaling pathways regulating pluripotency
drug metabolism by Cyp450
malaria
proteoglycans in cancer
chemokine signaling pathways
TNF signaling pathway
rheumatoid arthritis
Rap1 signaling pathway
melanogenesis
chemical carcinogenesis
cAMP signaling
calcium signaling
renin angiotensin system
bladder cancer
Hippo signaling pathway
0,00
1,00
2,00
3,00
4,00
AMPK-independent
AMPK- dependent

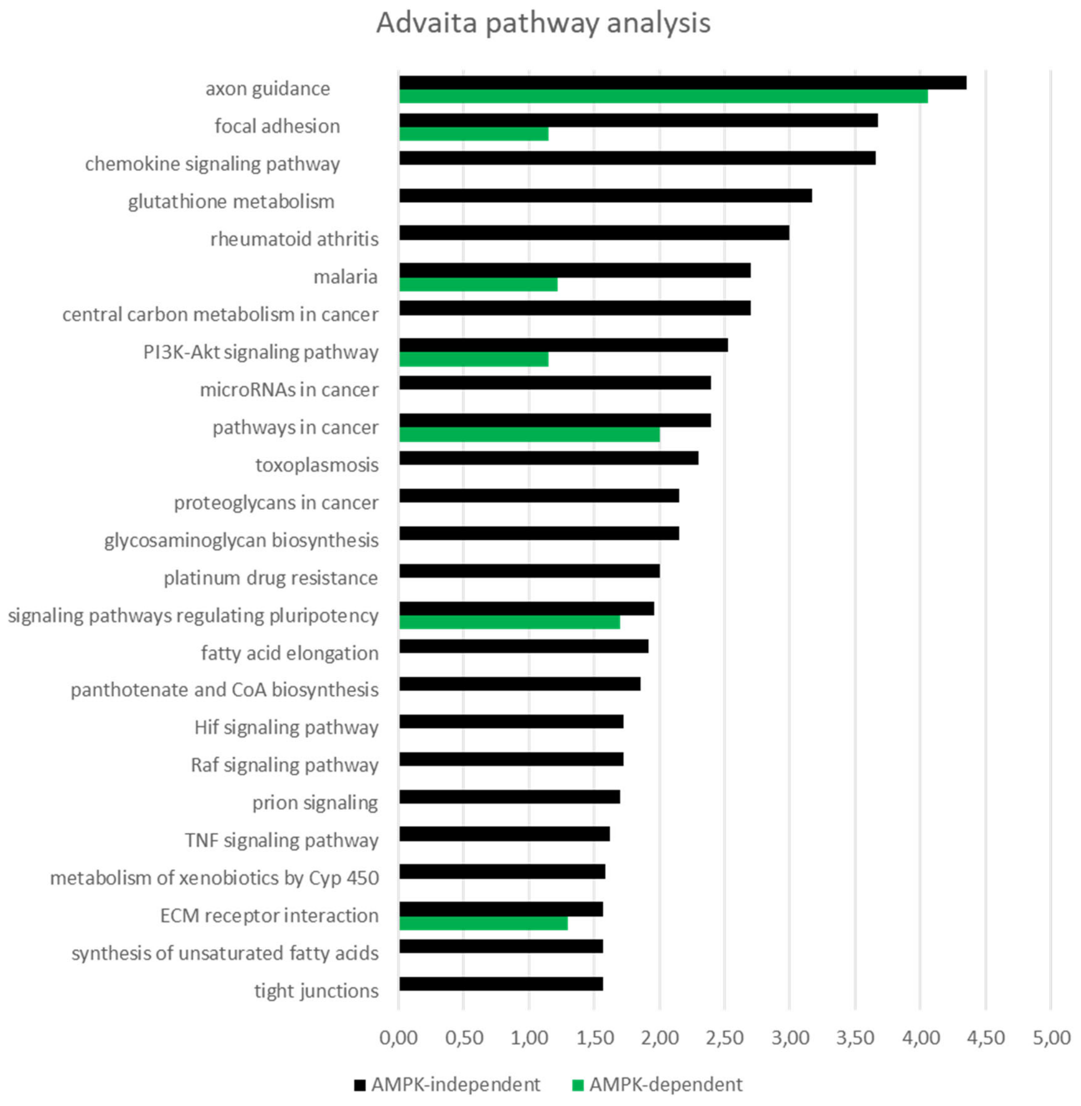

Supplemental Figure 1: Pathway analysis of only Nrf2-regulated and of AMPK and Nrf2-regulated genes using DAVID and Advaita analysis tools. (A) Pathways in bold were consistently found to be enriched by the two analysis tools, pathways in black were only susceptible to regulation by Nrf2, pathways in green are suggested to be under the control of both Nrf2 and AMPK. (B) Bar graph presentation.

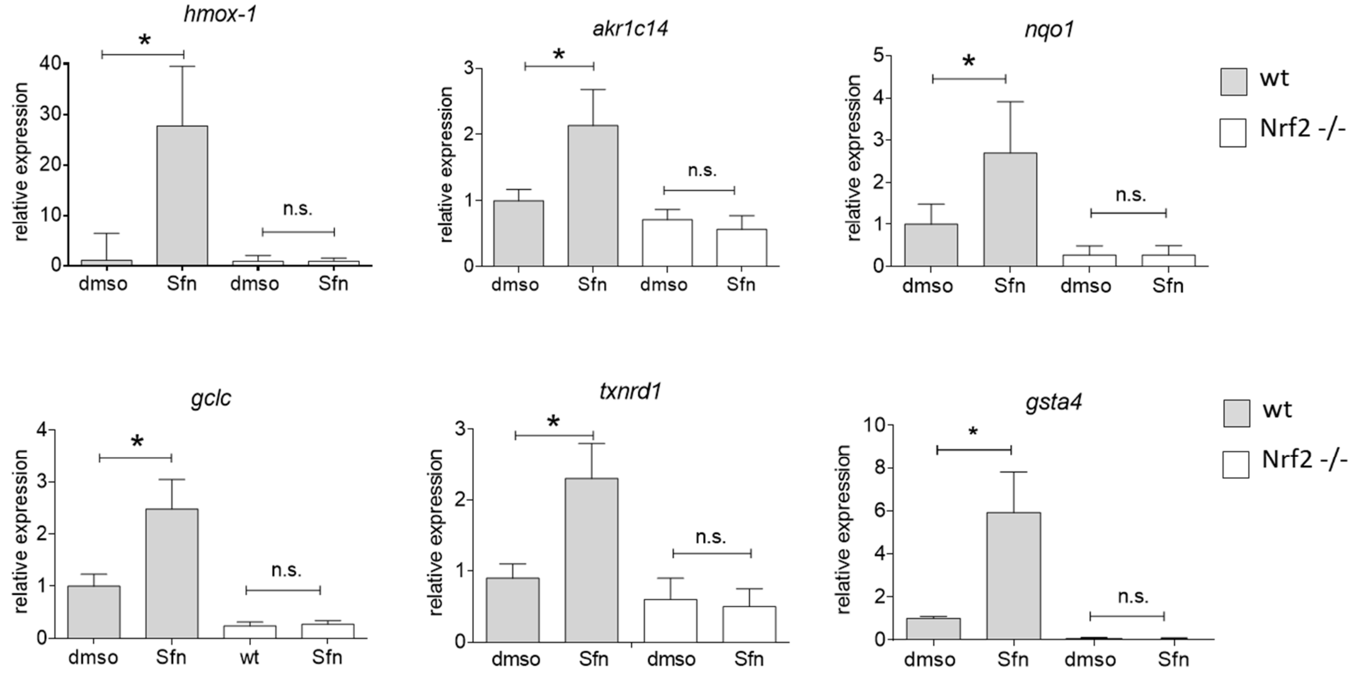

Supplemental Figure 2. The investigated genes are Nrf2-dependently induced by Sfn. Wt and Nrf2 -/- MEFs were treated with DMSO (0.1%) or sulforaphane (Sfn, 5 µM) for 4 h before RNA was isolated, reversely transcribed and subjected to qPCR analysis for *hmox1, akr1c14, txnrd1, gsta4, gclc* and *nqo1* as indicated (*hprt1* as reference gene). Bar graphs present the mean + 95% CI (* p<0.05, ANOVA, Tuckey)

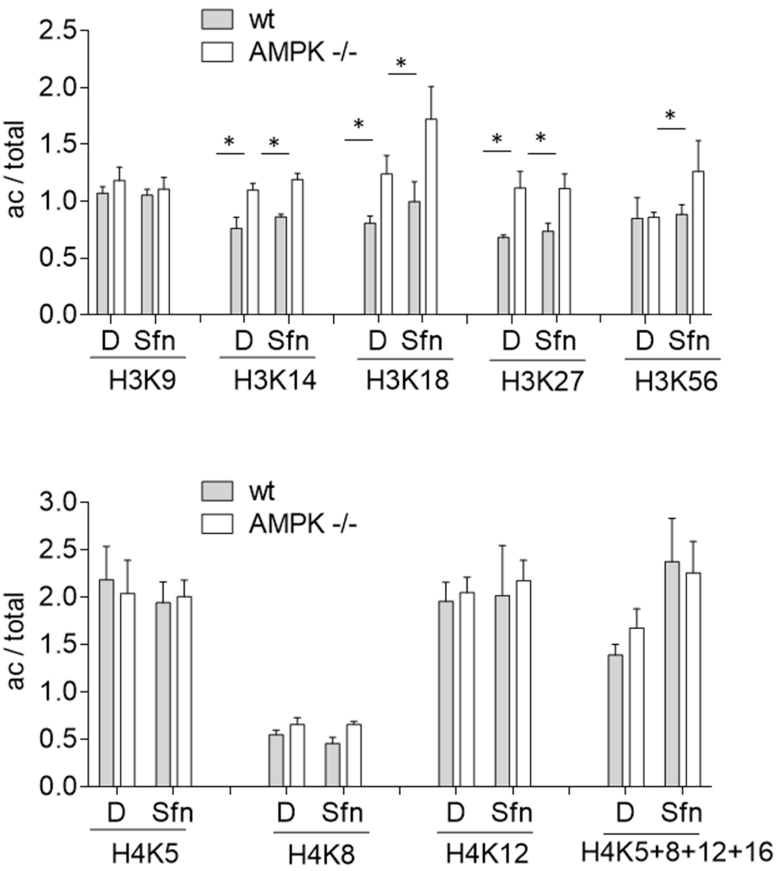

Supplemental Figure 3. AMPK-/- cells show increased global histone H3 acetylation. Histones 3 and 4 from wt and AMPK-/- MEFs were tested for acetylation at specific lysine residues by immunoblot. Compiled densitometric analyses are depicted (n=3, mean + SD; * $p<0.05$; ANOVA, Tuckey).

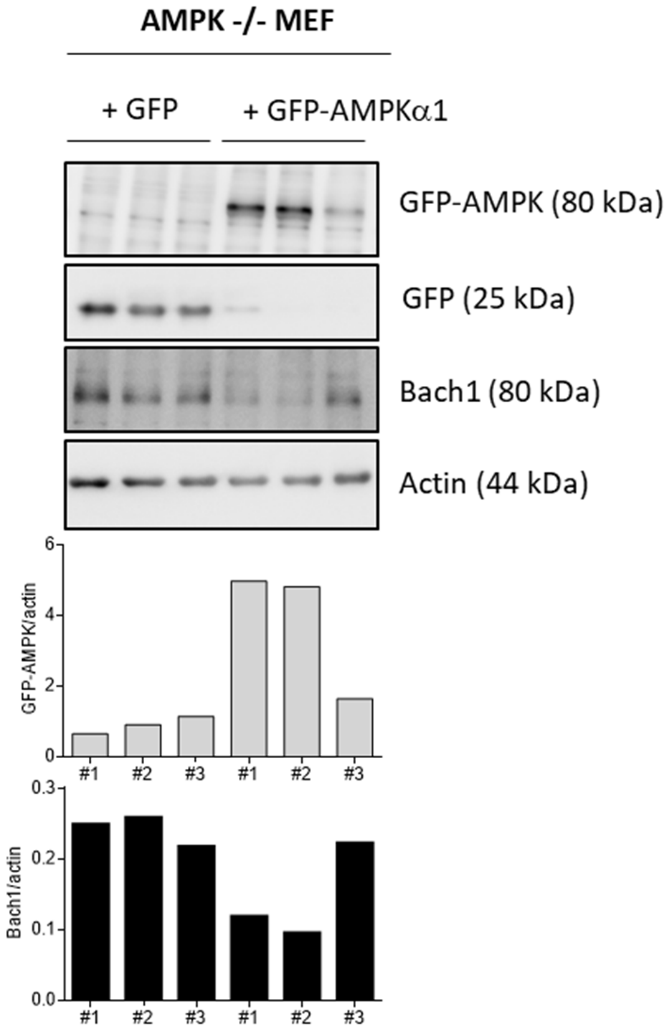

Supplemental Figure 4: Forced expression of AMPKα1 in AMPK-/- cells negatively correlates with Bach1 levels. AMPK-/- cells were transiently transfected with eGFP- or GFP-AMPKα1 expression plasmids (independent experiments #1-3). After 42 hours, cell lysates were probed for GFP, Bach1 and actin, respectively.

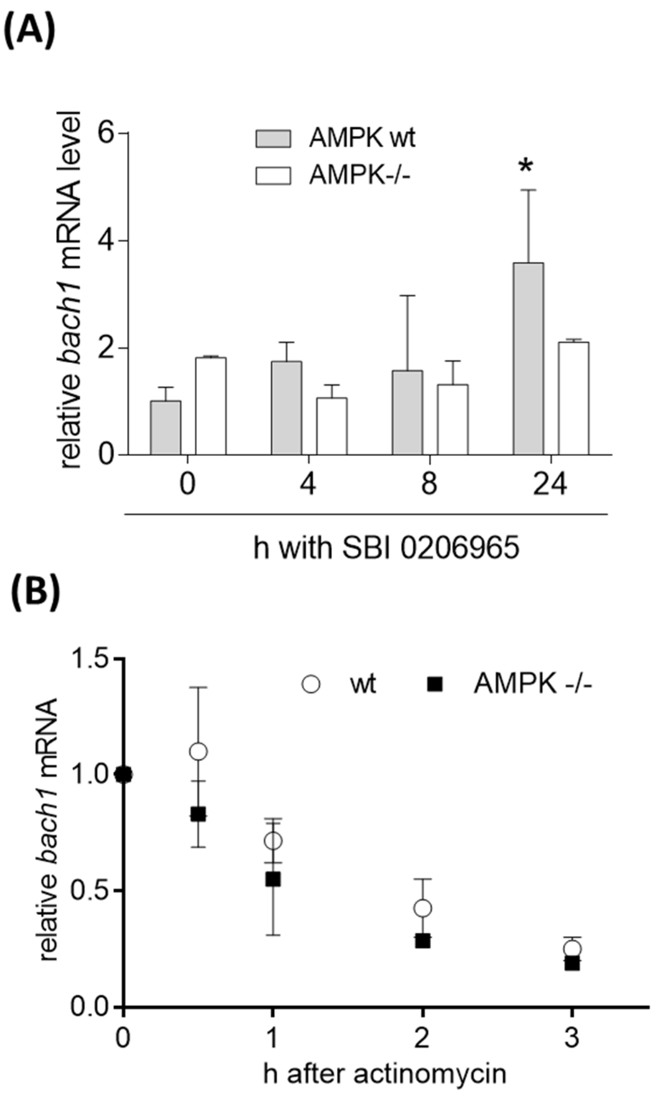

Supplemental Figure 5: *bach1* mRNA is AMPK-dependently suppressed and its half-life does not differ between wt and AMPK -/- MEFs. (A) Wt and AMPK-/- cells were treated with 0.1% DMSO or the AMPK inhibitor SBI0206965 (SBI, 30 µM) for the indicated time periods. Bach1 mRNA expression was then analyzed by qPCR (hprt1 as reference). (n=3, * $P \leq 0.05$ to t_0 (wt), ANOVA, Dunnett post-test). **(B)** Wt AMPK-/- cells were treated with actinomycin D (5 µM) for the indicated periods of time before mRNA was extracted, and *bach1* mRNA levels were determined by qPCR (*actinb* as reference gene, Qiagen) (n=2).
